# Diabetes Risk and Obesity in Food-Insecure Households in Rural Appalachian Ohio

**Published:** 2006-06-15

**Authors:** David H Holben, Alfred M Pheley

**Affiliations:** School of Human and Consumer Sciences, Ohio University; Department of Community and Rural Medicine, Virginia College of Osteopathic Medicine, Blacksburg, Va

## Abstract

**Introduction:**

In 2003, 11.2% of U.S. households were at some time food insecure; in 1999, when this study was conducted, 10.1% of U.S. households were at some time food insecure. A previous study of individuals from an Appalachian Ohio county suggested that food insecurity is associated with poorer self-reported health status. This larger study assesses the relationship of food security to clinical measurements of several chronic health risks among residents in six rural Appalachian Ohio counties.

**Methods:**

Data for this report are a subset of data gathered by surveys completed by 2580 individuals at community-based sites and by on-site, limited clinical health assessments conducted with a subsample of 808 participants. Descriptive statistics were calculated to describe the sample. Student *t* tests were used to compare measured BMI, diastolic blood pressure, total cholesterol, random blood glucose, HbA1c levels, and hemoglobin between individuals from food-secure and food-insecure households.

**Results:**

Our sample had about three times the level of food insecurity (with and without hunger) and more than seven times the level of food insecurity with hunger as the state population. Diastolic blood pressure, total cholesterol, random blood glucose, HbA1c, and hemoglobin did not differ by food security status (*P* > .05 for all); however, BMI was greater among individuals from food-insecure households, especially among women (*t*_1272 _= -2.0, *P *= .04), than among their food-secure counterparts. Obesity was greater among individuals from food-insecure households (48.1%) than among those from food-secure households (35.1%, *P* < .001).

**Conclusion:**

This study examines possible causes and consequences of food insecurity as it relates to chronic disease development. Further investigation is needed in this community  and in other Appalachian communities, as well as the United States, to determine relationships between food insecurity and chronic disease development and management.

## Introduction

Food security means having access, at all times, to enough food for an active, healthy life without resorting to using emergency food supplies, begging, stealing, or scavenging for food. Conversely, food-insecure individuals and families have limited access to or availability of food or a limited or uncertain ability to acquire food in socially acceptable ways (1). In 2003, 11.2% of U.S. households were at some time food insecure (2); in 1999, when this study was conducted, 10.1% of U.S. households were at some time food insecure (3). Overall, households in rural areas experienced more food insecurity than those in metropolitan areas (2,3).

*Healthy People 2010* includes the objective of increasing food security among U.S. households (4). Food insecurity can have negative consequences on health (5-9) and may cause physical impairments, psychological effects, and sociofamilial disturbances (10). For adults, food insecurity or insufficiency has been associated with lower dietary intakes of essential nutrients (11-14), fruits and vegetables (12,13), and milk and milk-based products (12) in addition to less-healthy diets (15). Over time, these suboptimal intakes could increase individuals' risk of developing diet-related chronic diseases (12). Food insecurity has also been associated with poor disease management in adults with chronic diseases, including diabetes (5-9,12,16-18).

Food insecurity among adults has been associated with being overweight or obese (19), especially among women (20-26). The relationship of obesity and food insecurity may be related to the low cost of energy-dense foods and reinforced by the pleasing taste of sugar and fat (27); however, food-insecure women do not seem to consume more high-fat, high-sugar, empty-calorie foods than their food-secure counterparts (28). Periods of overeating when food is available, including binge-like patterns of eating (13) or fluctuations in eating habits that promote a metabolic-adaptive response, may also account for overweight and obesity among adults from food-insecure households.

Pheley et al (6) found that individuals living in food-insecure households in a rural Appalachian Ohio community reported significantly worse functional health status than their food-secure counterparts, which was measured by the Medical Outcome Study Short Form-36 (SF-36) (29) and the 18-item U.S. Household Food Security Survey Module (FSSM) (30). To further investigate the health status and chronic disease risks of the community, we conducted a larger study. As part of this study, we assessed the relationship between food security and clinical measurements of several chronic health risks, including those that can contribute to obesity and diabetes.

The study area comprised some of the most impoverished counties of Ohio. Since 1980, rural regions in southern Ohio have lagged behind the rest of the nation in economic performance (31). Poverty is higher in rural Appalachia than in other regions, especially in southern Ohio (32). Because of the cost of developing roads, communication systems, public works facilities, and other infrastructure, health care delivery is often cost prohibitive. These limiting factors are present to an even greater extent in Appalachian areas than in other rural areas. Barriers to travel in inclement winter weather and flooding during early spring further complicate these factors.

## Methods

All study procedures were reviewed and approved by the Ohio University Office of Research Compliance before the collection of any data. Data for this article are a subset of data gathered to examine the relationship among self-described health status, access to and use of health care, and other personal characteristics of rural community-based participants. Specifically, we assessed the following variables: 1) self-reported household food security status; 2) self-reported diabetes; 3) BMI; 4) diastolic blood pressure; 5) total cholesterol; 6) random blood glucose; 7) hemoglobin A1c (HbA1c); and 8) hemoglobin. We also analyzed the relationship among these factors.

Household food security categories (food secure, food insecure with and without hunger, food insecure without hunger, food insecure with hunger [both moderate and severe]) were based on the FSSM. Overweight was categorized as a BMI of 25 kg/m^2^ or higher but less than 30 kg/m^2^, and obesity was categorized as a BMI higher than or equal to 30 kg/m^2^ (19). HbA1c levels less than 7% were considered to be within recommended ranges.

### Setting and participants 

This study was conducted in Athens, Hocking, Meigs, Perry, Pike, and Vinton counties in Ohio. These rural counties are in the northern Appalachian region of the United States (32,33). The U.S. Department of Agriculture's Economic Research Service has designated Athens and Vinton counties as *high poverty* because they have a poverty rate of 20% or more (34) and, based on the economic indicators of unemployment rates, per capita income level, and poverty (32), the Appalachian Regional Commission (ARC) has classified Athens, Meigs, Pike, and Vinton counties as *distressed*, which is the most severe economic level category. Perry and Hocking are categorized by the ARC as *transitional* counties, that is, counties that are at risk of becoming distressed. Table 1 provides demographic characteristics of the population in these counties.

Participants were recruited from the following community sites: 1) fairs and festivals; 2) food distribution programs for low-income families; 3) churches; 4) senior centers; 5) community action agencies and other programs offering services to families with limited resources; and 6) general sites, including grocery stores and shopping malls. From these convenience samples, 2580 adults aged 18 and older completed the study survey.

### Survey

From June through August 1999, research teams of 8 to 12 individuals set up tables and signage at the 31 study sites and invited people to complete a survey. All participants were provided a clipboard, a 12-page survey, and a pencil. Assistance was provided to participants who could not read or otherwise requested help in completing the questionnaire. When the participants completed the surveys, the surveys were reviewed on-site by project staff for completeness and legibility. The survey included demographic questions, information about the respondent's access to and use of health care, comorbid health conditions, and other validated measures (SF-36 [29] and FSSM [30]). Participants were provided a $5.00 gift certificate to a local grocery store as compensation for their time and effort.

### Clinical examination 

After they completed the survey, 808 participants (31.3%) agreed to undergo an on-site, limited clinical health examination. After these clinical health examination participants gave their written informed consent, they underwent an examination, regardless of their self-reported diabetes status. The components of the examination are described in Table 2 (38).

The devices used to measure biochemical indices were Clinical Laboratory Improvement Amendments (CLIA)-approved or waived (36). Each device was calibrated twice per day (once before morning sessions and again before afternoon sessions) according to manufacturer directions and commercial control solutions, except for the DCA 2000 (Bayer, Inc, Tarrytown, NY), which was calibrated after each series of 10 assays. Capillary blood collection and operation of the associated devices were conducted by second-year medical students under the supervision of the on-site physician. Each operator received 2 days of extensive training before the project began; staff evaluations and retraining were conducted throughout the study period.

Clinical health assessment participants were given an additional $5.00 grocery store gift certificate for their time and effort. Test results were provided by project staff to participants and to the physician or clinic identified by participants as their primary care provider. If the participant did not identify a primary care provider, the extra copy of the results was given to the individual. Participants were given the opportunity to speak with an on-site physician about any abnormal values. Although critical threshold values requiring immediate treatment for each of the screening tests were established before the study began, no individuals were identified who exceeded these criteria.

### Data management and analysis 

All survey data were entered electronically using a key-and-verify model to minimize data transcription errors and enhance data integrity. All analyses were conducted using the SPSS, version 10.0 (SPSS, Inc, Chicago, Ill). Descriptive statistics were calculated to describe the sample. Chi-square analysis was used to compare self-reported diabetes, obesity, and HbA1c levels greater than 7% between participants from food-secure and food-insecure households. Finally, Student *t* tests were used to compare measured BMI, diastolic blood pressure, total cholesterol, random blood glucose, HbA1c levels, and hemoglobin between individuals from food-secure and food-insecure households. *P* values (α = .05) are reported to communicate the strength of the relationships measured.

## Results

Table 3 describes the characteristics of the 2580 adults who completed the survey. Table 4 summarizes household food security status of our sample during the 12 months before sampling compared with the state- and national-level estimates of household food security status at the time of the study. It also shows that our sample had about three times the level of food insecurity (with and without hunger) and more than seven times the level of food insecurity with hunger as the state population. Of those participating, 1879 (72.8%) were from food secure households and 701 (27.2%) were from food insecure with and without hunger households. Of the total number of participants, 183 (7.1%) were from households classified as food insecure without hunger, and 518 (20.1%) were from households classified as food insecure with hunger. (Food insecurity with hunger included those classified as food insecure with moderate hunger [n = 248, 9.6%] and food insecure with severe hunger [n = 270, 10.5%].) 

Table 5 summarizes the clinical health examination results by food security status and by sex within the food security groups. Overall, the results were within recommended ranges; the exception was BMI, which exceeded healthy weight guidelines of 25 kg/m^2^ or less. Diastolic blood pressure, total cholesterol, random blood glucose, HbA1c, and hemoglobin did not differ by food security status (*P* > .05 for all); however, BMI was greater among participants from food-insecure households, especially among women (*t*_1272 _= -2.0, *P* = .04), than among their food-secure counterparts.

When stratified by sex, only BMI and HbA1c were significantly greater among women from food-insecure households than among those from food-secure households. For men, only hemoglobin levels were significantly greater among those from food-secure households than among those from food-insecure households.

Through chi-square analysis, we found that obesity was greater among individuals from food-insecure households (48.1%) than among those from food-secure households (35.1%, *P* < .001), and it increased monotonically as food insecurity worsened (from food secure to food insecure with hunger) (data not shown); 35.1% of obese individuals were from food-secure households. Of those obese individuals who were from food-insecure households, 43.4% were food insecure without hunger, 47.8% were food insecure with moderate hunger, and 52.4% were food insecure with severe hunger (Χ^2^ linear association, *P* < .001). Additionally, we found that individuals with an HbA1c level of higher than 7% (33.9%) were more likely (*P* = .053) to come from food-insecure households than respondents with HbA1c levels of less than 7% (22.5%) (data not shown).

Of the 2504 who noted their diabetes status, 298 (11.9%) reported having diabetes. People who reported having diabetes were significantly more likely to live in food-insecure households (37.9%) than in food-secure households (25.8%) (Χ^2^_1_ = 19.3, *P* < .001).

## Discussion

This study examines possible consequences of food insecurity as they relate to overweight, obesity, and chronic disease and highlights the need for further research on health status in the Appalachian region and the United States. The prevalence of food insecurity among this sample was more than 2.5 times the U.S. average rate (10.1%) in 1999 (3) and almost 3 times the Ohio average rate of household food insecurity at the time of the study (9.1%) (2).

A previous study suggested that even minimal levels of food insecurity are associated with poorer self-reported health status among individuals from an Appalachian Ohio county (6). Poor health status has also been associated with food insecurity in other studies (5,7-9). For adults, dietary quality can be negatively affected by food insecurity or insufficiency (11-13,15), and these suboptimal intakes could increase the public's risk of developing diet-related chronic diseases over time (12).

In this study, participants from food-insecure households had higher BMIs, rates of obesity, and self-reported rates of diabetes than those from food-secure households. No difference in food security status, however, was noted between diastolic blood pressure, total cholesterol, random blood glucose, HbA1c, and hemoglobin. This study is consistent with previous reports in which overweight or obesity have been associated with food insecurity among adults in rural and other areas of the United States (20-26). Women, unlike men, from food-insecure households had higher BMIs than those from food-secure households, a result which is consistent with national trends (20-26).

For our sample, the self-reported rate of diabetes (11.9%) was greater than the 2002 national average for adults aged 20 years and older (8.7%) (40); those reporting to have diabetes were more likely to reside in food-insecure than food-secure households. Likewise, individuals with HbA1c levels of higher than 7% were more likely to come from food-insecure households than those with levels of less than 7%. Although mean HbA1c levels were within normal ranges for both groups, previous reports cite that household food insecurity has been associated with poor disease management in adults with chronic disease (5,16-18).

Limitations of this study include our sampling strategy. Whereas data for the U.S. estimates are from national probability samples, our participants were a convenience sample and were not necessarily representative of the region. Our sampling process limited the generalizability of these data. A comparison of Census 2000 data (35) with our sample suggests our sample is more racially diverse, is somewhat overrepresented by women, includes fewer individuals without a high school education, and has a lower income than is typical of the counties where the sample was drawn. However, our data are consistent with trends previously reported and provide evidence of the possible health consequences of food insecurity.

Another limitation of this study was the use of nonfasting blood measurements; fasting measurements were prohibited by the nature of the sampling process. Although standards and reference values for laboratories differ, HbA1c and hemoglobin reference values for adults are generally nonfasting, and total cholesterol and random blood glucose are typically fasting, because values can be falsely increased without fasting. Even with this limitation, the total cholesterol and random blood glucose measurements were within recommended ranges.

This study examines possible causes and consequences of food insecurity as it relates to chronic disease development. Further investigation is needed in these and other Appalachian communities and throughout the United States to determine the relationship between food insecurity and chronic disease development and management. To improve outcomes associated with food insecurity (e.g., obesity, diabetes, poor disease management), dietetic, nutrition, medical, and community and public health professionals should incorporate food-security–related strategies into their practices (41,42). Furthermore, alleviating food insecurity in the United States seems contingent upon adequate funding and increased use of food and nutrition assistance programs; inclusion of food and nutrition education in all programs providing food and nutrition assistance to the public; and development of innovative programs to promote and support economic self-sufficiency of individuals, families, and households (43).

## Figures and Tables

**Table 1:** Demographic Characteristics of Six Ohio Counties, 2000^a^

**Characteristic**	**Athens**	**Hocking**	**Meigs**	**Perry**	**Pike**	**Vinton**	**Average of the Six Counties **
Women, %	51.1	50.2	51.4	50.3	51.2	50.2	50.7
White, %	93.5	98.9	97.7	98.5	96.7	98.1	97.2
Population aged ≥25 y with less than a high school education, %	17.1	22.0	26.8	21.1	29.9	29.3	24.4
Median household income, $	27,322	34,261	27,287	34,383	31,649	29,465	30,727

a Source: U.S. Census Bureau (35).

**Table 2:** Components of Limited Clinical Examinations of Study Participants Conducted in Six Rural Appalachian Counties, June–August, 1999

**Clinical Examination Component**	**Explanation**
Height	Measured to the nearest centimeter using a calibrated, portable stadiometer (36) (Seca Height Rod, Model 225, Hanover, Md)
Weight	Measured to the nearest half pound using a calibrated balance-beam scale (Health-O-Meter, Model 230PBD, Boca Raton, Fla) (36)
Body mass index	Calculated using weight/height^2^ (kg/m^2^)
Diastolic blood pressure	Measured to the nearest millimeter of mercury (mm/Hg) using standardized blood pressure techniques (37) with a calibrated, portable sphygmomanometer (Baumanometer, Desk Model 320, Copiague, NY)
Total cholesterol	Measured to the nearest milligram per deciliter (mg/dl) using a cholesterol meter; device calibrated twice each day using commercial control solutions according to manufacturer directions (Roche, Accu-Chek Instant Plus Monitor Model, Basel, Switzerland)
Random glucose	Measured to the nearest milligram per deciliter (mg/dl) using a glucose meter; device calibrated twice each day using commercial control solutions according to manufacturer directions (LifeScan, Inc, One-Touch Profile Blood Glucose Monitor, Milpitas, Calif)
HbA1c	Measured to the nearest .01%; device calibrated after every 10 assays using commercial control solutions according to manufacturer directions (Bayer, Inc, DCA Model 2000, Tarrytown, NY)
Hemoglobin	Measured to the nearest tenth of a gram per deciliter (g/dl) using a calibrated, portable meter (HemoCue, Inc, β-Hemoglobin Test System, Lake Forest, Calif)

**Table 3:** Characteristics of Survey Participants (N = 2580) in Six Ohio Counties, June–August, 1999

**Characteristic**	**Value^a^**
Mean age, y (SD)	44.7 (18.0)
Age, y	
<40	1,096 (42.5)
40-59	861 (33.4)
>60	597 (23.1)
Missing data (of total)	26 (1.0)
Women	1,706 (66.1)
White	2,277 (88.3)
Marital status – Married	1,433 (55.5)
Education – less than high school	414 (16.1)
Gross annual household income category, $	
<5,000	141 (5.5)
5,000 to 9,999	360 (14.0)
10,000 to 19,999	454 (17.6)
≥20,000	1,161 (45.0)
Missing data (of total)	464 (18.0)
Median income, $	22,000
Self-identified health status fair or poor	587 (22.8)

a All data are no. (%), unless otherwise noted.

**Table 4:** Household Food Security Status Among Sample and Ohio and U.S. Households, June–August, 1999

**Population**	**Food Secure**	**Food Insecure With and Without Hunger**	**Food Insecure Without Hunger**	**Food Insecure With Hunger**
Study sample, 1999, %^a^	72.8	27.2	7.1	20.1
Ohio households, 1999–2001, %^b^	91.9	9.1	6.3	2.8
U.S. households, 1999, %^c^	89.9	10.1	7.1	3.0

a U.S. Household Food Security Survey Module Questions (30) measure the 12 months before data collection, July–August, 1999.

b Source: Nord M, et al (39).

c Source: Andrews M, et al (3).

**Table 5:** Clinical Health Examination Results of Study Participants in Six Ohio Counties, June–August, 1999

**Clinical Examination Measure**	**Participants From Food-Secure Households**		**Participants From Food-Insecure Households**		**t** *_**df**_*	***P*** ** Value**

	**No.**	**Mean (SD)**	**No.**	**Mean (SD)**	
**Body mass index, kg/m^2^, n = 806**						
Men	230	28.8 (5.4)	54	29.2 (7.1)	−0.4_68_	.72
Women	390	29.1 (7.5)	131	30.8 (8.1)	−2.0_210_	.04
Total	620	29.0 (6.8)	185	30.3 (7.9)	−2.0_272_	.04
**Diastolic blood pressure, mm/Hg, n = 803**						
Men	231	80.8 (9.0)	54	79.6 (10.5)	0.9_283_	.43
Women	386	76.7 (9.9)	131	78.1 (12.4)	−1.2_189_	.24
Total	617	78.0 (10)	185	79.0 (12)	−0.3_263_	.74
**Total cholesterol, mg/dl, n = 808**						
Men	231	177.6 (33.0)	54	173.5 (33.4)	0.8_283_	.41
Women	391	187.0 (37.6)	131	184.8 (32.5)	0.6_520_	.53
Total	622	183.0 (36)	185	182.0 (33)	0.7_806_	.52
**Random blood glucose, mg/dl, n = 808**						
Men	231	106.9 (43.5)	54	111.3 (43.5)	−0.6_283_	.62
Women	391	101.8 (33.0)	131	109.4 (52.6)	−1.6_166_	.12
Total	622	104.0 (37)	185	110.0 (55)	−1.4_236_	.15
**HbA1c, %, n = 739**						
Men	194	5.53 (1.16)	51	5.63 (1.62)	−0.5_243_	.65
Women	371	5.29 (0.90)	122	5.57 (1.50)	−2.0_151_	.05
Total	565	5.40 (1.0)	173	5.60 (1.5)	−1.7_219_	.09
**Hemoglobin, g/dl, n = 808**						
Men	231	15.66 (1.58)	54	15.16 (1.72)	2.0_283_	.04
Women	391	13.64 (1.45)	131	13.84 (1.60)	−1.3_520_	.18
Total	622	14.40 (1.8)	185	14.20 (1.7)	−1.1_804_	.28
